# Impact of Hepatitis Virus on the Feasibility and Efficacy of Anticancer Agents in Patients With Hepatocellular Carcinoma in Phase I Clinical Trials

**DOI:** 10.3389/fonc.2019.00301

**Published:** 2019-04-18

**Authors:** Takafumi Koyama, Shunsuke Kondo, Toshio Shimizu, Yutaka Fujiwara, Chigusa Morizane, Yasunari Sakamoto, Takuji Okusaka, Noboru Yamamoto

**Affiliations:** ^1^Department of Experimental Therapeutics, National Cancer Center Hospital, Tokyo, Japan; ^2^Department of Hepatobiliary and Pancreatic Oncology, National Cancer Center Hospital, Tokyo, Japan

**Keywords:** hepatocellular carcinoma (HCC), phase I clinical trials, hepatitis virus, efficacy, feasibility

## Abstract

Chronic viral hepatitis is a risk factor for liver fibrosis and hepatocellular carcinoma (HCC). Patients with advanced HCC have limited effective therapeutic options and are considered potential candidates for early phase clinical trials of anti-cancer agents. The impact of chronic viral hepatitis on the efficacy of anticancer agents for patients with HCC in phase I trials (P-Is) still remains unclear and has not been reported. We retrospectively analyzed the outcomes of consecutive HCC patients in P-Is conducted in a single institute, focusing on chronic viral hepatitis. Of 85 patients enrolled in P-Is, 46 (54%) patients positive and 39 (46%) patients negative for chronic viral hepatitis showed no significant difference in clinical and laboratory variables and on the point of the best response based on the Response Evaluation Criteria in Solid Tumors (RECIST) criteria; moreover, the frequency of Grade ≥3 adverse events (AE) was not significantly different. The median time to treatment failure (TTF) and overall survival (OS) from the P-I enrolment were 2.0 and 13.7 months, respectively. No patient experienced reactivation of hepatitis B virus (HBV) or treatment-related death. Chronic viral hepatitis does not independently affect the outcomes of anticancer drugs. Advanced HCC patients with chronic viral hepatitis could be feasible for P-Is.

## Introduction

Hepatocellular carcinoma (HCC) is one of the most commonly found solid tumors and the fourth leading cause of cancer-related death worldwide (1). The incidence of HCC varies widely according to geographic location (2). This difference in distribution of HCC is probably related to regional variation in exposure to hepatitis virus and environmental pathogens. Over 50% of cases are due to underlying chronic viral hepatitis (hepatitis B or C virus infection) (1). The frequency of hepatitis B virus (HBV) carriers is relatively high in sub-Saharan Africa, the People's Republic of China, Hong Kong, and Taiwan (3). The prevalence of hepatitis C virus (HCV) has increased in the United States among individuals born between 1945 and 1965 (~2.5% or >2 million individuals, five times the rate among those born in other years) (4). Japan has one of the highest incidence rates of HCC associated with HBV or HCV infection (5, 6).

Until 2008, no effective therapy existed for patients with advanced-stage HCC or those for which local therapies failed. There has been a resurgence of interest and enthusiasm for systemic treatment of HCC after the emergence of data showing that treatment with the molecular targeting agent sorafenib (7) improved survival compared with that of best supportive care alone. A recent study showed that lenvatinib was non-inferior to sorafenib in patients with no prior systemic treatment for HCC (8). A survival benefit in the second-line treatment with regorafenib (9), ramucirumab (10), and cabozantinib (11) was demonstrated after the failure of sorafenib in HCC patients. Given the lack of established systemic treatment making survival significantly prolonged in patients with advanced HCC, early phase clinical trials are still needed to develop new treatment options for patients with advanced HCC.

Hepatic reserve, as indicated by Child-Pugh (C-P) classification, often dictated the therapeutic options (12). No prospective study has investigated the impact of hepatitis virus on treatment response and adverse events to investigational new drugs in phase I clinical trials (P-I) for advanced HCC patients. Advanced HCC patients with HBV infection might have a poorer prognosis than those with HCV infection (13). The A-P study showed that HCC patients with HBV infection who received sorafenib tended to have longer overall survival (OS) than patients who received a placebo (HR0.68) (14). Increasing data from subgroup analyses in phase III clinical trials suggested that patients with chronic hepatitis C as the etiology of their cirrhosis might show a better response to sorafenib than those with other underlying causes of cirrhosis (8, 15, 16). There were inconclusive data about how HBV or HCV infections affected the safety and efficacy of systemic therapy in patients with HCC during their participation in P-I. The present study was designed to examine the feasibility and efficacy of new anticancer drugs in patients with HCC who enrolled in P-I, from the perspective of HBV or HCV infection.

## Materials and Methods

### Data Collection

From July 2009 to August 2017, we retrospectively reviewed electronic medical records of consecutive patients with advanced HCC who enrolled in P-Is at the National Cancer Center Hospital, Tokyo (NCCH). This study was approved by the NCCH institutional review board and performed in compliance with our institutional guidelines.

Patients positive for anti-HCV antibody or HBV antigen (HBsAg) were considered to have HCC due to chronic viral hepatitis, whereas those without anti-HCV antibody nor HBsAg were considered to have HCC by another etiology (non-B/non-C).

All medical records were reviewed to summarize the patients' clinical characteristics, treatments, and outcomes based on the status of chronic viral hepatitis. The baseline clinical characteristics included age, gender, ECOG-performance status (PS), treatment history, spread from the primary site, number of metastatic sites and their locations, laboratory tests, and the C-P classification. The outcomes consisted of the date of P-I enrolment and progression, the date of death or loss to follow-up, the best response to therapy based on Response Evaluation Criteria in Solid Tumors (RECIST), and adverse events based on Common Terminology Criteria for Adverse Events (CTCAE) ver.4.

### Endpoints and Statistical Methods

The primary outcome of interest was the effect of chronic viral hepatitis on the efficacy and feasibility of the drugs on patients with HCC who enrolled in P-Is. We assessed the effectiveness through time to failure (TTF), OS, and the best response, and the feasibility through toxicity.

TTF was defined as the time from the start of P-I enrolment to discontinuation (as judged by the P-I investigators), based on clinical disease progression, treatment intolerance, toxicity, or death. OS was defined as the time from the P-I enrolment to death or the last day at which the patient was known to be alive; patients were censored if they were alive at the last follow-up. Kaplan-Meier product limit estimates were used to generate curves for TTF and OS. Univariate Cox proportional hazards models were fitted to test the effects of chronic viral hepatitis on TTF and OS.

Based on the status of chronic viral hepatitis, differences in the baseline clinical characteristics were evaluated using χ2 tests. The ratio of actual dose (AD) to recommended Phase 2 dose (RP2D) was calculated based on each trial result that was already reported. The distribution of dose ratio for each subgroup of patients was measured based on their hepatitis status and then evaluated using Wilcoxon tests.

The best response was assessed using the RECIST guidelines. Waterfall plots were created to illustrate the responses; subplots were also designed according to the drug classes. Treatment effects were estimated for each subgroup of patients based on their hepatitis status and evaluated using χ2 tests.

Toxicity data were assessed across trials in an aggregate fashion by considering the number of grade 3/4 events recorded. Patients who were taken off therapy because of secondary drug-related toxicity as well as those who died as a result of therapy were also analyzed. The percentage of patients with each type of adverse event was determined by dividing the number of patients with the adverse event by the number of patients based on the status of chronic viral hepatitis or C-P classification. Comparisons between the positive and negative status of chronic viral hepatitis and the correlation between C-P A and B were assessed across P-I studies. The analysis considered the total number of grade 3/4 events across trials with Fisher's exact tests based on the status of chronic viral hepatitis as well as C-P classification.

All tests were two-sided, and a *P* < 0.05 was considered statistically significant. All statistical analyses were performed using JMP software (version 13; SAS Inc., Cary, North Carolina, USA).

## Results

### Patient Characteristics

We identified 85 consecutive patients with HCC, who participated in P-Is at the NCCH. Forty-six patients were positive for chronic viral hepatitis, of which 26 were positive for HBsAg, 25 were positive for the anti-HCV antibody, and five were positive for both HBsAg and anti-HCV antibody, whereas 39 patients were negative for chronic viral hepatitis. Based on the status of chronic viral hepatitis, there were no significant differences in the baseline clinical characteristics, between the positive and negative groups (Table 1). There was also no difference in the distribution of dose Ratio of AD to RP2D between the positive and negative groups (*P* = 0.21).

**Table 1:** Patient characteristics based on the status of chronic viral hepatitis.

	**Chronic viral hepatitis**		
	**Positive *N* = 46**	**Negative *N* = 39**	
**Patient characteristics, n (%)**			***P***
Median age, years (range)	63.5 (42–77)	62 (38–80)	
<65	20 (44)	21 (54)	0.8
≥65	26 (56)	18 (46)	
Gender			0.35
Male	38 (82.6)	29 (74.3)	
Female	8 (17.3)	10 (25.6)	
ECOG performance status			0.22
0	34 (73.9)	24 (61)	
1	12 (26.1)	15 (38)	
Primary tumor (TMN UICC2017)			0.08
T0	0	3 (7.7)	
T1	0	0	
T2	21 (45.7)	21 (53.8)	
T3	11 (23.9)	5 (12.8)	
T4	14 (30.4)	10 (25.6)	
Ascites	8 (17.8)	8 (20.5)	0.75
Portal vein thrombosis	15 (33.3)	7 (17.9)	0.11
Portal vein invasion	17 (37)	10 (25.6)	0.26
Intrahepatic metastasis	43 (93.5)	36 (92.3)	0.83
Prior systemic treatments, median and range	1 (0-6)	2(0–8)	
Prior exposure to sorafenib	28 (60.9)	30 (76.9)	0.11
Child-Pugh			0.92
A	41 (89.1)	35 (89.7)	
B	5 (10.9)	4 (10.2)	

*HBV, Hepatitis B Virus; HCV, Hepatitis C Virus; ECOG, Eastern Cooperative Oncology Group; UICC, Union for International Cancer Control; RMH, Royal Marsden Hospital*.

### Survival and Efficacy

**Figure 1:**
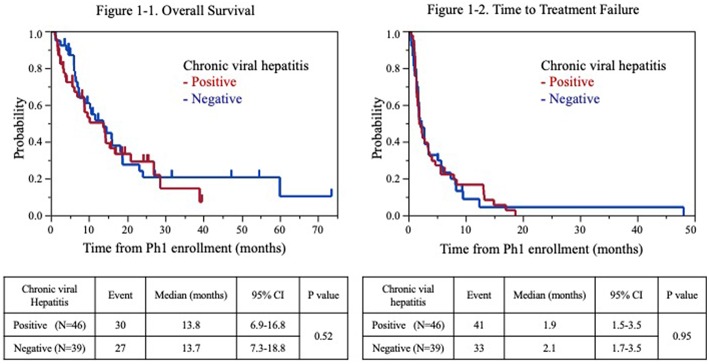
Kaplan-Meier estimates of overall survival (1-1) and time to failure (1-2) for patients with advanced hepatocellular carcinoma.

Fifty-seven patients (67%) had died at the time of the analysis. The TTF and OS from the P-I enrolment were 2.0 months [95% confidence interval (CI): 1.7–2.8] and 13.7 months (95% CI: 8.9–15.9), respectively. The median OS from the P-I enrolment for the populations positive and negative for chronic viral hepatitis was 13.8 months (95% CI: 6.9–16.8 months) and 13.7 months (95% CI: 7.3–18.8 months), respectively (Figure 1A). The 90-day mortality rate was 17.8 and 7.7% for patients positive and negative for chronic viral hepatitis, respectively. The survival rates of the population positive for chronic viral hepatitis at 6, 12, and 18 months were 69.7, 50.4, and 33.4%, respectively. The survival rates of the population negative for chronic viral hepatitis at 6, 12, and 18 months were 87.0, 51.6, and 37.8%, respectively. The TTF for the population positive and negative for chronic viral hepatitis was 1.9 months (95% CI: 1.5–3.5 months) and 2.1 months (95% CI: 1.7–3.5 months), respectively (Figure 1B).

All patients were evaluated for response based on the RECIST criteria. Based on the status of chronic viral hepatitis, between the positive group and negative group, there was no significant difference in the number of patients who participated in each trial, according to the drug class (Table 2).

**Table 2:** Mechanism of agent action in phase I trials enrolling patients in the present study based on the status of chronic viral hepatitis.

	**Trials**	**Chronic viral hepatitis**	
		**Positive *N* = 46**	**Negative *N* = 39**
**Mechanism of agent action, n (%)**			
FGFR inhibitor	2	1 (2.2)	1 (2.6)
GPC3 inhibitor	1	3 (6.5)	0
Immune checkpoint inhibitor	2	1 (2.2)	4 (10.3)
MEK inhibitor	1	1 (2.2)	1 (2.6)
Multiple tyrosine kinase inhibitor	4	12 (26.1)	8 (20.5)
Multiple tyrosine kinase inhibitor + cytotoxic drug	1	3 (6.5)	1 (2.6)
Multiple tyrosine kinase inhibitor + HDAC inhibitor	1	7 (15.2)	3 (7.7)
Multiple tyrosine kinase inhibitor + STAT3 inhibitor +	1	1 (2.2)	0
PDGFR α inhibitor	1	1 (2.2)	1 (2.6)
PI3K/Akt pathway inhibitor	2	1 (2.2)	3 (7.7)
Stemness kinase inhibitor	2	2 (4.3)	4 (10.3)
STAT3 inhibitor	2	6 (13)	5 (12.8)
TEM-1 inhibitor	1	0	3 (7.7)
Virus/Vaccine	2	7 (15.2)	8 (20.5)

*FGFR, Fibroblast Growth Factor Receptor; GPC3, Glypican 3; MEK, Mitogen-Activated Protein Kinase Kinases; HDAC, Histone Deacetylase; STAT3, Signal Transducers and Activator of Transcription 3; PDGFRα, Platelet-derived Growth Factor Receptor alpha; PI3K, Phosphatidylinositol-3 kinase; TEM-1, Tumor Endothelial Marker-1*.

**Figure 2:**
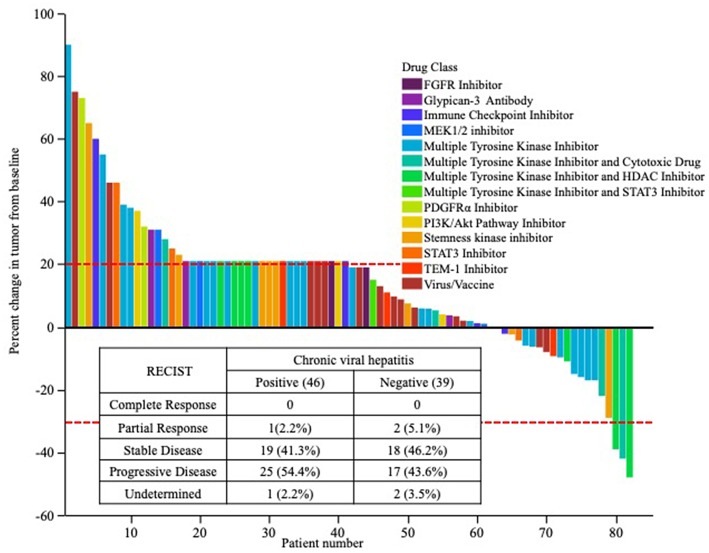
Objective tumor responses according to each drug type used in the phase I trials.

There was no significant difference between patients positive (Partial response; 1, stable disease; 19, progressive disease; 25, and not evaluated; 1) and negative for chronic viral hepatitis (Partial response; 2, stable disease; 18, progressive disease; 17, and not evaluated; 2) (*P* = 0.65) (Figure 2). The best responses of patients according to drug class are shown in Figure 2.

### Safety

We observed no treatment-related mortality or HBV reactivation. The anti-viral drug Entecavir was prescribed for all patients with chronic HBV infection during the P-Is. Ten patients who received an angiogenesis inhibitor (e.g., Multiple Tyrosine Kinase inhibitor) experienced Grade (Gr) 3 hypertension. An increase in Gr 3 AST or ALT was observed in five patients positive for chronic viral hepatitis during the P-Is (Table 3). Fisher's exact test for the frequency of Gr ≥3 adverse events based on hepatitis virus infection status as well as C-P classification revealed no significant difference (Table 3).

**Table 3:** Adverse event based on the status of chronic viral hepatitis and Child-Pugh classification.

	**Chronic viral hepatitis**			**Child-Pugh classification**		
	**Positive *N* = 46**	**Negative *N* = 39**		**A *N* = 76**	**B *N* = 9**	
**Adverse event (Grade 3** **≥), n (%)**			***P***			***P***
Neutropenia	3 (6.5)	0	0.052	3 (3.9)	0	0.41
Lymphopenia	0	1 (2.6)	0.21	1 (1.3)	0	0.64
Thrombocytopenia	2 (4.3)	0	0.11	2 (2.6)	0	0.50
Nausea/vomiting	2 (4.3)	1 (2.6)	0.65	3 (3.9)	0	0.41
Fatigue	2 (4.3)	0	0.27	1 (1.3)	0	0.63
Diarrhea	1 (2.2)	0	0.27	1 (1.3)	0	0.64
Skin rash	2 (4.3)	0	0.91	2 (2.6)	0	0.50
Hand foot syndrome	1 (2.2)	0	0.27	1 (1.3)	0	0.64
ALT increased	1 (2.2)	0	0.27	1 (1.3)	0	0.64
AST increased	5 (10.7)	0	0.064	4 (5.2)	1 (11.1)	0.16
Bilirubin increased	1 (2.2)	0	0.91	1 (1.3)	0	0.50
GGT increased	2 (4.3)	1 (2.6)	0.87	3 (3.9)	0	0.34
ALP increased	1 (2.2)	1 (2.6)	0.65	1 (1.3)	0	0.28
Hypertension	8 (17)	2 (5.1)	0.070	9 (11.7)	1 (11.1)	0.95
Other	4 (8.8)	3 (7.8)	0.83	8 (10.4)	0	0.24

*ALT, Alanine Transferase; AST, Aspartate Transferase; GGT, Gamma- Glutamyl Transferase; ALP, Alkaline Phosphatase*.

The reasons for discontinuing the P-I treatment of patients positive for chronic viral hepatitis were disease progression (*n* = 33, 84.6%), toxicity (*n* = 4, 10.2%), and patient refusal (*n* = 2, 5.1%). Toxicity included Gr 3 infection (1), Gr 3 fatigue (1), Gr 3 hand-foot syndrome (1), and Gr 4 neutropenia (1). The reasons for discontinuing the P-I treatment of patients negative for chronic viral hepatitis were disease progression (41 patients; 89.1%), toxicity (4 patients; 8.7%), and patient refusal (1 patient; 2.2%). Here, toxicity included Gr 3 increased gamma-glutamyl transferase (GGT) (1), Gr 3 skin rash (1), Gr 3 hypertension (1), and Gr3 nausea (1).

## Discussion

This is the first report of the impact of chronic viral hepatitis on the efficacy and feasibility of anticancer agents for patients with HCC in P-Is. We conducted a retrospective study on 85 consecutive patients with advanced HCC who enrolled in P-Is independent of the status of chronic viral hepatitis. Patients with chronic viral hepatitis comprised 54.1% of all patients included in the analysis. There was no evidence that positive or negative chronic viral hepatitis affected OS, TTF, and disease control rate. There was no significant difference in the frequency of Gr ≥3 AE, between the positive group and negative group. No patient showed reactivation of hepatitis virus, and no treatment-related mortality was observed.

To date, in most P-I that enrolled patients with all types of solid tumors, patients with chronic viral hepatitis have been excluded. Hence, there are limited clinical data on the effect of chronic viral hepatitis on the efficacy and feasibility of drugs in patients enrolled in P-Is. Recently, the results of a phase I study of nivolumab for patients with advanced HCC (CheckMate 040) showed that efficacy and feasibility were similar in patients with or without chronic viral infection (17). On the other hand, liver function might be related to feasibility in P-Is. In the Phase I study of lenvatinib for patients with advanced HCC, the maximum tolerable dose (MTD) was different between C-P A group (12 mg once daily) and C-P B group (8 mg once daily). The most common Gr 3 toxicities included hypertension in C-P A and hyperbilirubinemia in C-P B (18). Patients with hepatic impairment have higher plasma concentration of lenvatinib and may have increased risk of adverse events compared with patients without hepatic compromise. We have to consider hepatic function when we enroll HCC patients in P- I treatment, which is primarily excreted through the liver (12).

Pre-clinical data from mouse models of cancer with hepatitis virus infection are mostly unavailable because hepatitis viruses do not infect mice. Although mouse models do exist to mimic viral hepatitis, it was difficult to predict whether P- I treatment would cause severe complications such as hepatocyte destruction owing to drug effect on infected hepatocytes.

There is a difference in the mechanism of carcinogenesis between HBV and HCV. Liver inflammation and hepatocyte proliferation driven by host immune responses in hepatocytes with HBV infection are recognized as driving forces of liver cell transformation (19). In the absence of a cytopathic effect in hepatocytes infected with HBV, the oncogenic role of HBV might involve a combination of direct and indirect effects of the virus during the multistep process of liver carcinogenesis (20). The higher level of damage to hepatocytes with chronic HCV infection could be explained by the imbalance in the microenvironments and cytokines of livers infected with the HCV, leading to increased inflammation and cell turnover, which ultimately causes cirrhosis (21). A meta-analysis of three phase III randomized controlled trials demonstrated higher survival benefit to patients positive for HCV than for HBV (15).

This study has some limitations. The first is that it is a retrospective single-center study, which limited the inclusion of some variables because of lack of data. Second, the etiology of an abnormal liver function test is challenging to define in an HCC study population because an abnormal liver function test result may be related to factors such as tumor progression or drug-related toxicity. Third, we did not monitor HBV DNA level and HCV RNA level for each case. Most of the trials we analyzed in this article were conducted before the confirmation by recent studies that effective viral suppression could reduce the risk of HCC (22). Finally, a small number of patients were treated in different trials and at varying doses and achieved different outcomes, which precludes more robust statistical analyses and warrants caution when interpreting our findings. Furthermore, we were unable to conduct an effective multivariate analysis, given the small sample size.

Chronic viral hepatitis did not independently affect the efficacy and feasibility of the P-I treatment, although it was one of the most significant factors that contributed to the pathogenesis of HCC. The poor outcomes and limited treatment options for advanced HCC patients emphasize the need for new approaches. P-Is that are based on an enrichment design such as specific histologic characteristics and particular biomarkers are associated with a higher probability of clinical benefit than those that are not (23). Advanced HCC patients, independent of the status of chronic viral hepatitis, could be feasible for P-Is and might clinically benefit from treatment in the P-Is.

## Ethics Statement

This study was performed under the Declaration of Helsinki with regard to ethical principles for research involving human subjects. The NCCH institutional review board approved this study's design, and the study was performed under its guidelines.

## Author Contributions

TK and SK contributed to the conceptualization, methodology, investigation, writing the original draft, and revision the manuscript. TS, YF, CM, YS, TO, and NY contributed to the investigation and revision of the manuscript.

### Conflict of Interest Statement

TS received consulting fees from Takeda, The Consortium on Harmonization of Institutional Requirements for Clinical Research (CHAIR), Hong Kong and HKSAR; and funds for research expenses from Bristol-Myers Squibb, Daiichi-Sankyo, Takeda-Millennium, FivePrime, PharmaMar, 3D Medicine Symbio Pharmaceuticals, and Chordia Therapeutics. TO received consulting fees from Eli Lilly, Dainippon Sumitomo Pharama, Taiho Pharamaceutical Co and BMS and funds for research expenses from Eli Lilly, Eisai, Dainippon Sumitomo, Taiho Pharamaceutical Co, BMS, Kowa K.K., Kyowa Hakko Kirin Co., Merk Serono Co, Ono Pharamceutical Co., Ono Pharmaceutical Co., Novartis, Pfizer Jana Inc., Bayer Yakuhin, Ltd., Chugai Pharmaceutical Co. Ltd., Yakuruto Honsya Co. Ltd., Asrtra Zeneka K.K. and Zeria Pharmaceutical Co. Ltd., and honoria from Eli Lilly, Eisai, Dainippon Sumitomo Pharama, Taiho Pharamaceutical Co, BMS., Ono Pharmaceutical Co., Novartis, Pfizer Jana Inc., Bayer Yakuhin, Ltd., Chugai Pharmaceutical Co. Ltd., Yakuruto Honsya Co. Ltd., Asrtra Zeneka K.K., Zeria Pharmaceutical Co. Ltd., Daiichi Sankyo Co. Ltd., Nippon Chemifa Co. Ltd., EA Pharma Co. Ltd., Fujifilm RI Pharma Co. Ltd., Celgene k.k., Teijin Pharma CO. Ltd., MSD, Shire, Abbvie and Takeda Pharmaceutical Co. Ltd. NY received consulting fees from Eisai, Takeda, Otsuka, Boehringer Ingelheim and Cimic and funds for research expenses from Quintiles, Astellas, Chugai, Esai, Taiho, BMS, Pfizer, Novartis, Daiichi-Sankyo, Bayer, Boehringer Ingelheim, Kyowa-Hakko Kirin, Takeda, ONO and Janssen Pharma and honoraria from BMS, Pfizer, AstraZeneca, Eli Lilly, ONO and Chugai. The remaining authors declare that the research was conducted in the absence of any commercial or financial relationships that could be construed as a potential conflict of interest.
